# New Appliances and Things Medical

**Published:** 1896-11-21

**Authors:** 


### NEW APPLIANCES AND THINGS MEDICAL.

**[We shall be glad to receive, at our Office, 28 & 29, Southampton Street, Strand, London, W.C., from the manufacturers, specimens of all new preparations and appliances which may be brought out from time to time.]**

### HANSON'S SANITARY FLUID.

(Hanson and Co., 19, St. Dunstan's Hill, London, E.C.)

The above antiseptic fluid, a sample of which has recently been forwarded us, appears to us, after a somewhat extended trial, to be a first-class domestic disinfectant and deodorizer. Being neither corrosive, nor poisonous in ordinary quantities, it is a far safer disinfectant than many that are in general use. It has a clean, fresh odour, and may be used for washing animals and destroying all sorts of parasites. A powder incorporated with the above antiseptic fluid is prepared by the same firm, and for many purposes offers greater advantages than the liquid form. The fluid, when diluted with 40 parts of water, appears to possess antiseptic qualities sufficient for all ordinary purposes.

